# Ultraviolet Germicidal Irradiation and Its Effects on Elemental Distributions in Mouse Embryonic Fibroblast Cells in X-Ray Fluorescence Microanalysis

**DOI:** 10.1371/journal.pone.0117437

**Published:** 2015-02-23

**Authors:** Qiaoling Jin, Stefan Vogt, Barry Lai, Si Chen, Lydia Finney, Sophie-Charlotte Gleber, Jesse Ward, Junjing Deng, Rachel Mak, Nena Moonier, Chris Jacobsen

**Affiliations:** 1 Department of Physics & Astronomy, Northwestern University, Evanston, Illinois, USA; 2 X-ray Science Division, Advanced Photon Source, Argonne National Laboratory, Argonne, Illinois, USA; 3 APS Engineering Support Division, Advanced Photon Source, Argonne National Laboratory, Argonne, Illinois, USA; 4 Chemistry of Life Processes Institute, Northwestern University, Evanston, Illinois, USA; Irvine, UNITED STATES

## Abstract

Rapidly-frozen hydrated (cryopreserved) specimens combined with cryo-scanning x-ray fluorescence microscopy provide an ideal approach for investigating elemental distributions in biological cells and tissues. However, because cryopreservation does not deactivate potentially infectious agents associated with Risk Group 2 biological materials, one must be concerned with contamination of expensive and complicated cryogenic x-ray microscopes when working with such materials. We employed ultraviolet germicidal irradiation to decontaminate previously cryopreserved cells under liquid nitrogen, and then investigated its effects on elemental distributions under both frozen hydrated and freeze dried states with x-ray fluorescence microscopy. We show that the contents and distributions of most biologically important elements remain nearly unchanged when compared with non-ultraviolet-irradiated counterparts, even after multiple cycles of ultraviolet germicidal irradiation and cryogenic x-ray imaging. This provides a potential pathway for rendering Risk Group 2 biological materials safe for handling in multiuser cryogenic x-ray microscopes without affecting the fidelity of the results.

## Introduction

X-ray fluorescence microscopy (XFM) and tomography at synchrotron radiation facilities has a long history [1–3] in providing unprecedented sensitivity for low-damage studies of metal and ion distributions in cells and tissues, as discussed in recent reviews [4–9]. While much successful work has been carried out on room temperature dehydrated specimens, it has long been shown in electron [10, 11] and x-ray [12–14] microscopies that rapidly-frozen hydrated specimen preparations viewed at cryogenic temperatures offer excellent structural preservation. Cryo microscopy methods also provide superior preservation of trace element concentrations, as has been demonstrated in electron microprobe analysis [15–19] and in XFM [20–22].

Many XFM studies employ specimens originated from human organs, tissues and cell lines; since they may contain potentially infectious bloodborne pathogens such as hepatitis B (HBV) and human immunodeficiency viruses (HIV), they are often classified as Risk Group 2 (RG2) materials. Research using RG2 materials has to follow certain biosafety requirements (such as those enforced in the USA by the Occupational Safety & Health Administration), including the use of personal protective equipment for researchers, working with RG2 materials only in designated Biosafety Level 2 (BSL2) areas, and decontaminating all contacted instruments and areas afterwards. These practices are generally easy to follow in a designated BSL2 wet lab.

Consider now the case of a cryo-scanning microscope operating in a vacuum environment, such as the Bionanoprobe (developed by Xradia Inc., which is now Carl Zeiss x-ray Microscopy Inc.). This scanning fluorescence microprobe operates at the Life Sciences Collaborative Access Team (LS-CAT) beamline at the Advanced Photon Source (APS) at Argonne National Laboratory (ANL) [22]. The Bionanoprobe has a cryo transfer system with samples loaded in cartridges that are robotically transferred to and from the microscope scanning stage, and the ability to hold four samples in addition to the one under study. While such a failure has not happened yet, imagine the case of having a frozen hydrated RG2 specimen be “lost” inside the vacuum chamber, yet land on a cryo-cooled surface. If the vacuum chamber of the system were to be suddenly vented to air, the cryo-cooled samples would warm up and melt, and one would have hydrated RG2 materials within a complicated microscope system. In this case, decontaminating the entire system becomes necessary before resuming routine operations. Given the mechanical complexity involved this would be a daunting task.

While such a scenario might be regarded as unlikely, low probability events must be considered when designing safe protocols. It is therefore important to consider ways of rendering originaly-RG2 materials into a biologically non-infectious state so that subsequent handling can be according to BSL1 practices. Conventional chemical fixation has been recognized as one such way to deactivate most living organisms [23–25]. Thus one possibility is to employ chemical fixation before biological cells and tissues are rapidly frozen. However, chemical fixation is known to cause changes in the chemical makeup of cells [26–30], and cellular structures [31–35]. Due to the slow penetration of fixatives into cells and tissues as well as subsequent changes in membrane permeability, various cellular ions could be leached, lost or relocated, thus complicating investigations aimed at understanding their biological functions. In comparisons of chemically fixed versus cryopreserved mouse fibroblasts, Matsuyama *et al.* have shown that chemical fixation leads to significant changes in the distribution of diffusible or weakly bound elements such as Ca, K and Fe, with smaller but noticeable changes observed for tightly bound elements such as Cu and Zn [20]. Thus, to image elemental compositions and distributions as close as possible to the living state in cryo-scanning x-ray fluorescence microscopy, chemical fixations should be avoided.

We have therefore explored a different approach to render frozen hydrated cells into a non-infectious state: the use of ultraviolet germicidal irradiation (UVGI). UV irradiation in the 240–280 nm wavelength range leads to dimerization of thymine molecules on DNA [36]; this alteration in nucleic acids blocks transcription, thereby preventing cell replication. UVGI has been widely acknowledged as an efficient measure to deactivate potentially infectious microorganisms [37], including rhinovirus [38], *Mycobacterium tuberculosis* [39–41], *Bacillus cerus* and *anthracis* [42, 43], and *Serratia marcescens* [41]. It has been frequently applied in various confined and open spaces, such as biological safety cabinet interior surfaces [44], hospital rooms and public buildings [45, 46]. It has also been used to sterilize consumable products in food industries [47]. Therefore we explore in the present study whether the employment of UVGI could be a good approach to decontaminate frozen hydrated specimens, and more importantly to investigate whether it will alter elemental content and distribution in biological cells as revealed by XFM imaging.

## Results

Because frozen hydrated specimens must be maintained at temperatures below about 135 K to avoid ice crystal formation, we have undertaken tests to see whether UVGI is effective to deactivate microorganisms in a liquid nitrogen bath. Compared to UV irradiation conducted in the air, the actual irradiation level received by samples immersed in liquid nitrogen bath might be reduced due to possible UV absorption by liquid nitrogen. Two experiments were designed to evaluate such absorption by using “fastcheck” strips as the indicator of UV irradiation level (as described in Materials and Methods). In one experiment, one set including 3 “fastcheck” strips were placed in air, while another set including 3 “fastcheck” strips were placed under 3 cm of liquid nitrogen at the same distance from the UV light source; three strips in both sets were then irradiated for 5, 10 or 20 minutes, respectively. Compared to the corresponding strips in the air, the strips immersed in liquid nitrogen bath didn’t develop any color until they were taken out from liquid nitrogen bath and warmed to room temperature, and the indicated UV irradiation level was much reduced. This could be due to a combination of two factors: absorption of UV light in liquid nitrogen, and also reduction in induced chemical change to the “fastcheck” strips when cold (for example, in x-ray experiments we know that while primary bond breaking is not reduced at cryogenic temperatures, secondary effects such as mass loss are greatly reduced [48], so it could be that “fastcheck” color changes are also reduced when UV exposure is done at liquid nitrogen temperature). Nevertheless the indicated irradiation level in the liquid-nitrogen-immersed “fastcheck” strips were still linearly correlated with UV exposure time. Thus, these strips can be still considered to be used as a relative indicator of UV irradiation in liquid nitrogen bath. The second experiment was designed to find out whether the “fastcheck” strips placed in the gas phase of liquid nitrogen (0 cm depth) or at different depths of liquid nitrogen bath (1 cm, 3 cm, and 6 cm respectively) developed differently under the same exposure time and the same distance from the UV light source. We observed no difference among these four strips, suggesting that UV absorption by 3 cm liquid nitrogen bath is negligible, which is consistent with previous reports [49, 50]. As a result, we conclude that the irradiation level received in 3 cm depth of liquid nitrogen bath is roughly equivalent to that received in the air under the same irradiation condition.

**Fig 1:**
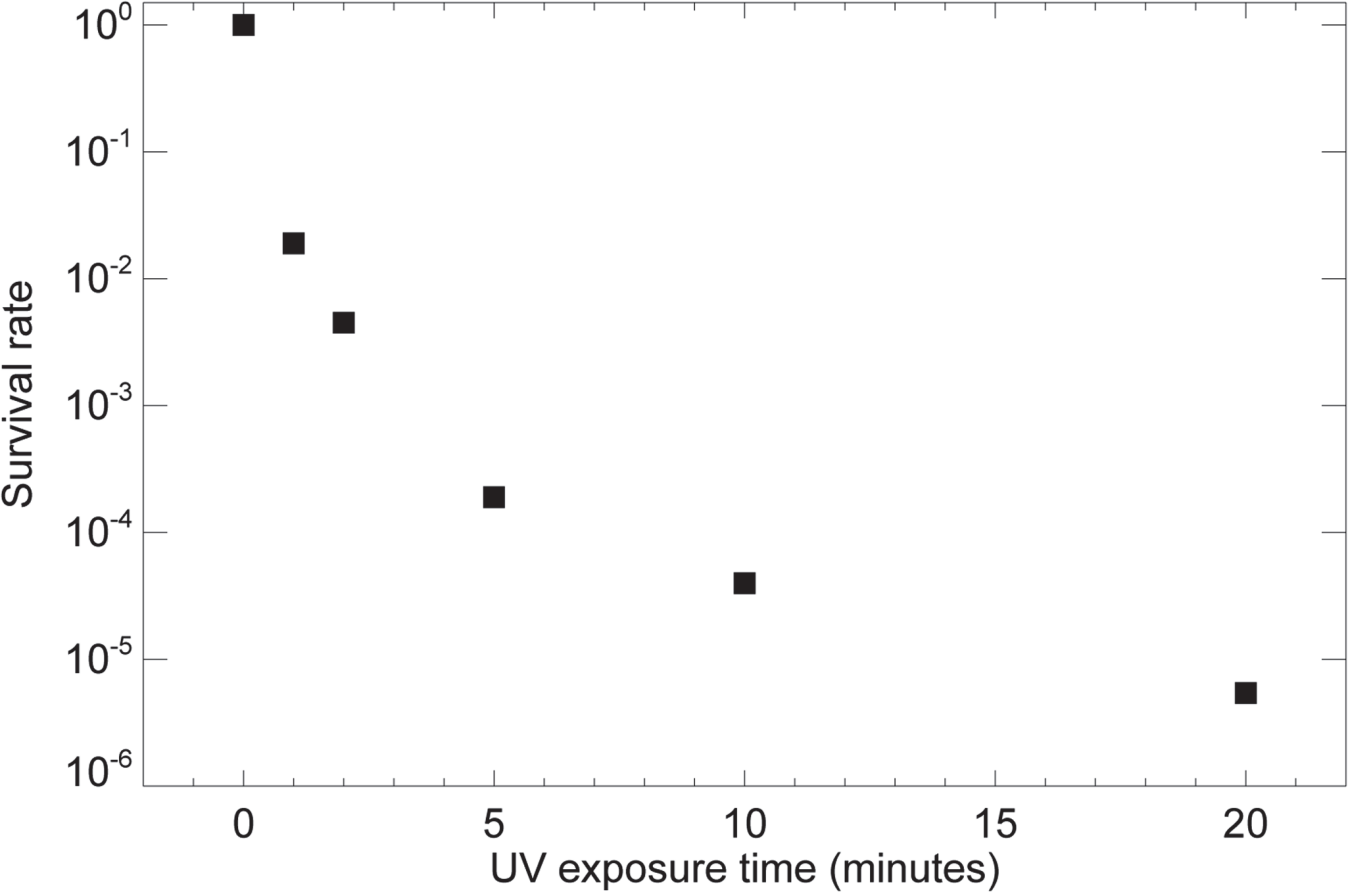
The survival rate of T7 bacteriophage under prolonged UV irradiation. About 60 million T7 bacteriophages in 3 *μ*l solution were dropped on Si_3_N_4_ windows and frozen by liquid-nitrogen-cooled liquid ethane, and then subjected for 0, 1, 2, 5, 10 and 20 minutes of ultraviolet germicidal irradiation while immersed in 3 cm of liquid nitrogen bath. A standard plaque assay was carried out to calculate the survival rate under each exposure, with three independent experiments being averaged to arrive at each point.

We used a bacterial virus (T7 bacteriophage) to establish the duration of UV exposure required to reduce infectivity. T7 bacteriophage is a double-stranded DNA virus capable of infecting susceptible bacterial cells. Although distinct differences exist between T7 bacteriophage and potentially infectious agents such as HIV and HBV associated with RG2 materials, they share essential structures including a protein shell and a DNA/RNA core. As illustrated in Fig. 1, when a droplet of about 6 × 10^7^ T7 bacteriophages was plunge frozen but not subject to UV exposure, nearly 100% survived, indicating that plunge freezing itself does not affect infectivity. When UV exposures of 5 or 10 minutes were used, all but about 10^−4^ of bacteriophages lost the capability of forming plaques in host cells. Although every virus is different, it has been reported that most infectious viruses are completely destructed at the energy intensity of 5 to 50 mJ/cm^2^ [49, 50]. With our current UVGI setting, the minimum irradiation level required to kill T7 bacteriophages was 5 minutes (equivalent to 50 mJ/cm^2^), which was quite comparable to that reported [49, 50]. Thus we believe 10 or 20 minutes of UVGI (equivalent to about 100 to 200 mJ/cm^2^) should be sufficient enough to kill most infectious agents associated with human-originated cell lines and tissue sections. While one often sees a simple inverse exponential relationship of survival rate with UVGI, thus leading to data falling on a straight line on a log (survival rate) versus exposure curve [51], we see in this experiment a slower-than-exponential decrease in survival rate for which we lack a good explanation.

**Fig 2:**
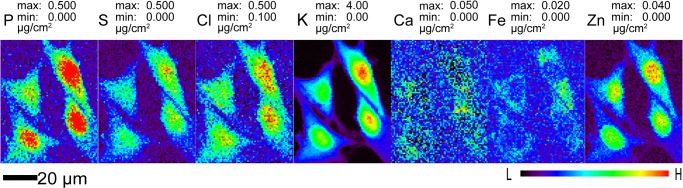
Representative elemental maps of frozen hydrated mouse embryonic fibroblast cells (S1 File). Cells previously cryofixed by plunge freezing were scanned using 10 keV X rays in the cryojet x-ray fluorescence microscope at APS beamline 2-ID-D at 100 K. These scans have 800 nm pixel size and a per-pixel dwell time of 1 second. Data are available in Supporting Information file S1.zip.

Having demonstrated that UVGI can deactivate T7 bacteriophages even when immersed in liquid nitrogen, we then carried out experiments to examine whether the contents and distributions of key biological elements were affected in eukaryotic cells after ultraviolet exposure. For these studies, we used adherent mouse embryonic fibroblast cells, which though they are RG1 materials, have UV response mechanisms and sensitivity [52] similar to those of human cells such as MCF-7 breast cancer cells [53]. As described in Materials and Methods, these mouse fibroblasts were grown on silicon nitride windows (Si_3_N_4_) which were then plunge-frozen in liquid ethane and examined at a temperature of approximately 100 K using cold nitrogen gas flow from an Oxford cryojet system. In x-ray fluorescence microscopes, an energy-dispersive detector is used to record the x-ray fluorescence emission spectrum as the cell is scanned through the beam, allowing one to quantitate the distributions of several chemical elements simultaneously as shown in Fig. 2. These images show that diffusible ions (Cl, K, Ca) are nicely contained within the cell membrane in cryo preparations.

**Fig 3:**
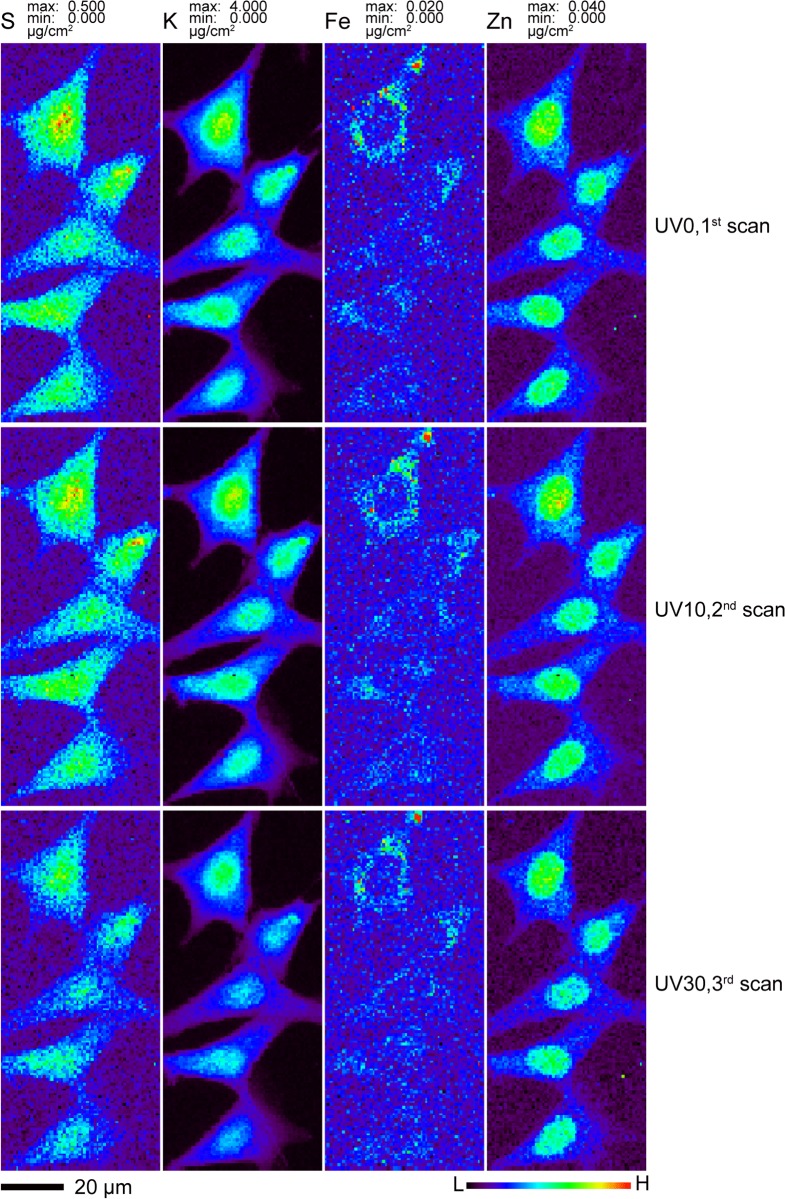
Effects of UVGI, cryo transfer and cryo-scanning on the elemental contents and distributions of frozen hydrated mouse embryonic fibroblast cells. Cryofixed cells were first imaged in the cryojet x-ray fluorescence microscope at 100 K without any prior ultraviolet exposure (UV0, the first scan). They were then removed while being maintained at cryogenic conditions, exposed to 10 minutes of UVGI while immersed in liquid nitrogen, and cryogenically imaged again (UV10, the second scan) before an additional 20 minutes of UV exposure were used and followed by a third scan (UV30 for 30 minutes accumulative exposure time). The x-ray scan parameters were identical to those used to produce the images of Fig. 2 as described in Materials and Methods. The elemental image for the same element was scaled to the same maximum and minimum values among these scans for direct visual comparison. Data are available in Supporting Information files S2.zip, S3.zip, and S4.zip for 0, 10, and 30 minutes of UV exposure, respectively.

**Fig 4:**
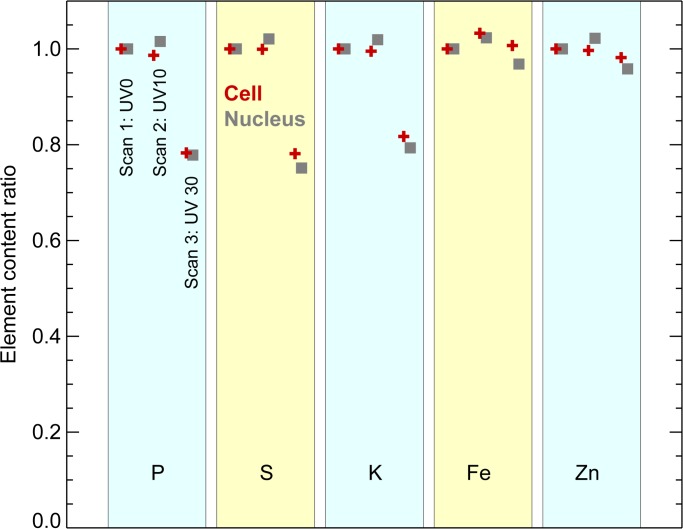
Elemental content in frozen hydrated mouse embryonic fibroblast cells, based on the images shown in Fig. 3. Elemental content within entire cells (red crosses) and their nuclei (gray squares) are plotted as fractions against the content in the first, non-UV-irradiated scan. These measurements were made from four of the five cells shown in Fig. 3; the next-to-top cell was excluded due to the difficulty in outlining its area for analysis. The slight decrease observed for the measured content of lighter elements (P, S, K) levels is consistent with a possible explanation of ice or frost accretion on the specimen between the second and third scan, leading to increased self-absorption of these lower energy fluorescent x-rays. This was further supported by the nearly unchanged ratio of elemental contents in the cell’s nucleus versus the entire cell among these three scans.

Our first test was to look for changes in the total contents and distributions of various elements as the same cell preparation was examined with XFM under frozen hydrated state before and after different doses of UVGI (Figs. 3). One area containing five cells on Si_3_N_4_ windows was scanned at 100K for the first time without any UVGI (UV0, first scan). This window was then unloaded for 10 minutes of UVGI under liquid nitrogen and uploaded for the second scan (UV10, second scan). After the second scan was completed, it was unloaded for an additional 20 minutes of UVGI and uploaded for the third scan (UV30, third scan). No apparent difference in cell size and morphology were observed among these three scans; all cells stayed intact without visible breakage and shrinkage. The distribution patterns of various elements including P, S, Cl, K, Ca, Fe, Cu and Zn remained nearly identical among the three scans under this resolution. Two-dimensional elemental mapping of four representative elements S, K, Fe and Zn was shown in Fig. 3. S and K maintained their homogeneous distribution, while Fe was primarily localized in cytoplasm and Zn was more concentrated in the nucleus than in the cytoplasm, consistent with other observations [20]. To look for any possible changes in total elemental content, four out of these five cells and their corresponding nuclei were subjected for ROI (region of interest) analysis. The total elemental contents within these ROIs were calculated and plotted as average fractions to that of UV0. As shown in Fig. 4, the average elemental contents between first and second scans remained almost the same for all elements at both the nucleus and the whole cell level. Although the third scan showed a 19–25% decline for lighter elements P, S and K, the decline was no more than 5% for heavier elements such as Fe and Zn. Self-absorption of x-ray fluorescence emission in an 5 *μ*m ice layer would account for a signal reduction to 74% of the previous signal for P and 81% for S, which is close to the observed reductions to 76% and 80% respectively, so ice accretion between the UV10 and UV30 scans might explain this element-dependent signal change. When the percentage of elemental content in the nucleus versus the whole cell was calculated, the difference between UV0 and UV30 scans was negligible for all measured elements, further supporting this explanation since an accreted ice layer would affect nuclear and cytoplasmic regions in the same way.

**Fig 5:**
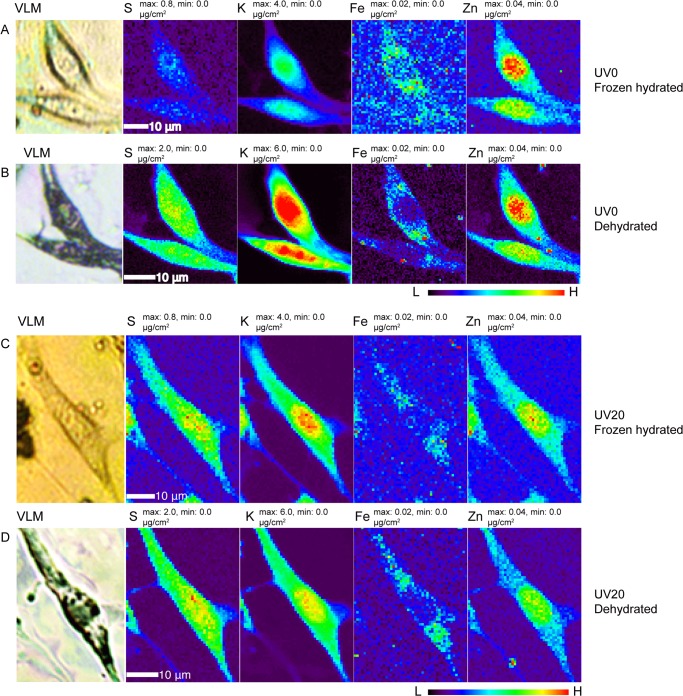
Comparison of elemental distribution maps of four elements (S, K, Fe, Zn) in frozen hydrated cells (rows A and C) as well as freeze-dried counterparts (panels B and D), with (rows C and D) and without (rows A and B) 20 minutes of ultraviolet germicidal irradiation (UVGI) under liquid nitrogen prior to frozen hydrated imaging. Visible light micrographs (VLM) of frozen hydrated (rows A and C) and freeze dried (rows B and D) cells show that the cells suffered similar degree of shrinkage after x-ray cryo-scanning and freeze drying, whether or not UVGI was used. Please note the maximum and minimum concentration values for the light elements S and K in row A were scaled to be the same as that in row C, but different from that in rows B and D. Scan parameters were the same as used for Figs. 2–4. Data are available in Supporting Information as files S5.zip and S6.zip for dehydrated cells exposed to UVGI for times of 0 and 20 minutes, respectively, and as files S7.zip and S8.zip for frozen hydrated cells exposed to UVGI for times of 0 and 20 minutes, respectively.

We have shown above (Figs. 3 and 4) that cellular ions are immobilized in the matrix of a frozen hydrated specimen maintained at cryogenic temperature, so that their distributions and concentrations are unchanged when subject to UVGI. However, most x-ray fluorescence microscopy studies are carried out using dehydrated specimens imaged at room temperature (there are only a few instruments available worldwide with cryo transfer capabilities [20–22]). We therefore attempted to investigate what effects, if any, our proposed sterilization treatment has on cells that are subsequently dehydrated and warmed for room temperature studies. We know for example that polymer films maintained at cryogenic temperatures will exhibit chain scission at radiation doses of 10^7^ Gray, though with little mass loss [48]. We also know that regions of cryogenic frozen hydrated cells exposed to doses of about 10^10^ Gray can show significant mass loss when they are subject to freeze-drying [13]. We therefore made two different frozen hydrated cell preparations, and exposed one to 20 minutes of UVGI while the control sample was not. We then made x-ray fluorescence microscopy images of both preparations in a frozen hydrated state (again involving a radiation dose of about 10^7^ Gray) using the cryojet microscope (rows A and C in Fig. 5). Afterwards, both preparations were removed from the XFM, subjected to the standard freeze-drying procedure described in Materials and Methods, and x-ray fluorescence micrographs were acquired of the room temperature dehydrated cells (rows B and D in Fig. 5). The results show that x-ray irradiation followed by freeze drying leads to the same pattern of elemental distributions but some overall morphological change (approximately 20% shrinkage); some of this change might have been caused by the reactivity of x-ray irradiation products in both organic material and in ice, as the warming used in freeze-drying allowed for some chemical reactivity beyond what was possible while the irradiated sample was maintained at cryogenic temperatures [13]. However, this shrinkage did not appear to depend on whether or not UVGI was used prior to the first frozen-hydrated x-ray image scan. In particular for the lighter elements, the elemental concentration *increased* in the dehydrated specimens relative to the frozen hydrated specimens; we again attribute this to much reduced fluorescence self-absorption due to the removal of most water/ice upon freeze drying. Most importantly, we did not observe any large-scale *decrease* in elemental concentrations that might have otherwise been due to the loss of metal-containing organic functional groups during the freeze-drying process, or the UVGI process.

For all scans shown here, we have made the raw data available as HDF5 files (www.hdfgroup.org/HDF5/) in Supporting Information.

## Discussion

As demonstrated extensively in the field of electron microscopy and more recently in x-ray microscopy, cryogenically prepared frozen hydrated cells imaged at cryogenic temperatures show greater fidelity to living cells in morphology, structure, and chemical composition when compared to chemically fixed and dehydrated counterparts. Unfortunately, cryopreserved RG2 materials must be considered to be potentially infectious. We have shown here that UVGI in liquid nitrogen bath is sufficient to deactivate T7 bacteriophages (Fig. 1), and more importantly that the application of UVGI has negligible effects on the distributions of metals in frozen hydrated samples as shown in Figs. 2–5. (UV irradiation might lead to changes in metal oxidation states, but we did not carry out x-ray near-edge absorption spectroscopy studies to see whether this is the case). This provides a potential specimen treatment that could be considered to avoid biological contamination of sophisticated cryogenic x-ray microscopes [20–22].

We chose a UVGI procedure of first producing a frozen hydrated cryogenic specimen, and then irradiating that specimen with ultraviolet light while immersed in liquid nitrogen of no more than 3 cm depth. One alternative procedure might be to carry out UVGI on initially-living cells before cryopreservation, which would exclude more instruments and space to come in contact with RG2 materials; however, this might involve complications of specimen heating under ultraviolet irradiation, as well as biochemical reactivity of radiolysis products with possible morphological and elemental distribution changes. In addition, ultraviolet sterilization is easily compromised by any sort of overlying material [43] such as a thicker overlying culture medium. In contrast, cryogenic conditions largely halt the reactions of radiolytical products and thus might prevent additional unintended effects beyond sterilization, and furthermore liquid nitrogen immersion maintains cells at a steady cryogenic temperature during irradiation. Parmegiani *et al.* have shown that UVGI can be used to sterilize liquid nitrogen used for the preparation and storage of human oocytes [49, 50]. Together, these factors led us to attempt UVGI of samples under liquid nitrogen.

For both frozen hydrated and dehydrated samples, we first attempted parallel comparison among cell samples irradiated with different doses of UVGI, aiming to find any dose dependent effects on elemental contents and distributions. It was however inconclusive due to wild cell-to-cell variasions resulting from different cell size, morphology and unsynchronized cell cycle phases. To exclude such preexisting variations, we changed to examine the same cells before and after different doses of UVGI under frozen hydrated state, or to examine the same cells before and after freeze drying from samples receiving different doses of UVGI. Our results indicate that UVGI has no detectable effects on elemental concentrations and distributions when examined with x-ray fluorescence microscopy under both cryogenic and room temperature conditions (Figs. 3–5). This suggests that UVGI immediately after cryofixation could be adopted as a routine practice to render RG2 materials to be handled as RG1 materials, without compromising elemental analysis by x-ray fluorescence microscopy. Finally, while x-ray microprobes have been used to study both monolayer cell preparations and tissue sections, here we tested cryogenic UVGI on cells only; with tissue sections, one would have to consider issues of UV absorbance in thicker sections.

## Materials and Methods

### Preparation and UVGI of T7 bacterophages

Approximately 6 × 10^7^ plaque forming units (pfu) of T7 bacteriophage in 3 *μ*l freshly prepared phage extract were dropped onto the center of Si_3_N_4_ windows and manually dipped into liquid-nitrogen-cooled liquid ethane using tweezers. After freezing, the windows were transferred from liquid ethane to liquid nitrogen, and laid flat with the bacteriophage facing up within a container and with about 3 cm of liquid nitrogen above. The container was then placed into a Millipore UV Sterilizer equipped with 4 × 8 W germicidal lamps (Sylvania No. G8T5, 253.7 nm wavelength), where it received UV irradiation for 0, 1, 2, 5, 10, or 20 minutes. For treatment duration beyond 10 minutes, UV irradiation was paused every 10 minutes to replenish the liquid nitrogen level back to 3 cm. After UVGI, the cryogenic windows were directly dropped into 297 *μ*l of Luria-Bertani (LB) broth at room temperature and subjected for a standard plaque assay procedure (T7 select manual from EMD Millipore, www.emdmillipore.com). The survival rate was calculated as the number of survival phages in every treatment divided by the number of phages in original 3 *μ*l phage solution. The entire experiment was repeated three times with similar results. To convert UV exposure time to an estimated UV irradiation level, UV “fastcheck” strips from UV Process Supply Inc. (catalog number N010-002) were used. By comparing the color changes on strips to the reference chart provided by the manufacturer, ultraviolet irradiation level can be estimated for any particular UV irradiation settings. Due to the extremely low temperature, the color development of “fastcheck” strips immersed in liquid nitrogen was significantly retarded and reduced compared to strips placed in room temperature air. As a result, the actual irradiation level received under liquid nitrogen was estimated by measuring the level received in the air under the same setting minus any UV absorption by 3 cm of liquid nitrogen as discussed above in Results.

### Cell culture and cryogenic sample preparation

Mouse embryonic fibroblast cells NIH/3T3, originally purchased from ATCC (www.atcc.org, Catalog number CRL1658), were cultured at 37°C in a 95% humidified incubator with 5% CO_2_ in Dulbecco’s Modified Eagle’s Medium ((Life Technologies, Grand Island, NY 14072, USA) supplemented with 10% fetal bovine serum (HyClone, Thermo Fisher Scientific, Inc., Pittsburgh, USA), 1% non-essential amino acids and 1% L-glutamine (Life Technologies, Grand Island, NY 14072, USA), following standard protocols [20, 54]. When about 80% confluence was reached, cells were passaged onto a new well of 6-well culture plate with a density of 6 × 10^4^ cells per well in 3 ml of culture medium. For cells to be imaged by XFM, Si_3_N_4_ windows (Silson Ltd., catalog number SiRN-5.0-200-1.5-200, 200 nm thick, 1.5×1.5 mm window area) were washed with distilled water followed by 2 mins soaking in 70% ethanol and 2 mins soaking in 100% ethanol, and then dried and taped to the bottom of a well in a 6-well culture plate, with the flat side up. After 20 minutes of UV sterilization, cells were seeded on Si_3_N_4_ window-containing wells and grew to 50–70% confluence.

The Si_3_N_4_ windows with cells grown on them were carefully detached from the well and rinsed two times by gently dipping into an Eppendorf tube containing 1 ml of Tris-glucose buffer (TG buffer, containing 261 mM glucose and 9 mM acetic acid in 10 mM Tris buffer, pH 7.4) [20]. Excess liquid on the backside of the window (non-cell side) was removed by blotting with filter paper. The windows were then attached to the tweezers supplied with a FEI Vitrobot Mark IV plunge freezer. Plunge freezing was carried out by plunging cells into liquid-nitrogen-cooled liquid ethane following the manufacturer’s instructions with chamber set at a temperature of 30°C, 100% humidity, and with the following blotting parameters: blot time = 2 s, blot force = 0 mm, and blot total = 1. The frozen cells on Si_3_N_4_ windows were then transferred from liquid ethane to liquid nitrogen for storage, from which they could be retrieved for ultraviolet irradiation as described above or x-ray imaging experiments.

### X-ray fluorescence microscopy

For cells imaged at room temperature, previously plunge-frozen samples were placed into the precooled vacuum chamber of EMS turbo freeze drier K775 and dried with the following cycles: 2 h at -120°C, 1 h from -120°C to -80°C, 3 h at -80°C, 1 h from -80°C to -50°C, 2 h at -50°C, 5 h at 25°C as suggested by the manufacturer. The cryofixation quality of frozen hydrated cells was monitored by observation using a 20x objective in a Nikon 50i light fluorescent microscope equipped with an Instec CLM77K cryo stage, while freeze-dried specimens were examined using a Leica DMXRE microscope. Frozen hydrated cells were scanned at around 100 K by an Oxford cryojet-equipped x-ray fluorescence microscope located at beamline 2-ID-D of APS at Argonne National Laboratory. Freeze-dried cells were scanned by x-ray fluorescence microscope located at beamline 2-ID-E at APS. Cells of interest were raster scanned with 0.8 *μ*m pixel size and a 10 keV beam with a focus diameter of about 0.5 *μ*m. The x-ray induced secondary fluorescence at each scan position was measured using an energy dispersive silicon drift detector (Vortex EM, SII Nanotechnology), and then fitted and quantified by comparison against the fluorescence signals from NBS standards 1832 and 1833 using MAPS software [55, 56].

## Supporting Information

As Supporting Information, the following HDF5 image files (www.hdfgroup.org/HDF5/) contain the elemental concentration maps used to produce the Figures in this paper.

S1 Image_1.zip This image contains the representative elemental maps of frozen hydrated mouse embryonic fibroblast cells shown in Fig. 2. (ZIP)

S2 Image_1.zip This image contains the fluorescence maps of frozen hydrated cells obtained before UltraViolet Germicidal Irradiation (UVGI), which are shown as the UV0 images in Fig. 3. (ZIP)

S3 Image_1.zip This image contains the fluorescence maps of frozen hydrated cells obtained after 10 minutes of UVGI, which are shown as the UV10 images in Fig. 3. (ZIP)

S4 Image_1.zip This image contains the fluorescence maps of frozen hydrated cells obtained after an additional 20 minutes of UVGI, which are shown as the UV30 images in Fig. 3. (ZIP)

S5 Image_1.zip This image contains the fluorescence maps of dehydrated cells obtained before UVGI, which are shown as the UV0 Dehydrated images in Fig. 5. (ZIP)

S6 Image_1.zip This image contains the fluorescence maps of dehydrated cells obtained after 20 minutes of UVGI, which are shown as the UV20 Dehydrated images in Fig. 5. (ZIP)

S7 Image_1.zip This image contains the fluorescence maps of frozen hydrated cells before UVGI, which are shown as the UV0 Frozen hydrated images in Fig. 5. (ZIP)

S8 Image_1.zip This image contains the fluorescence maps of frozen hydrated cells after 20 minutes of UVGI, which are shown as the UV20 Frozen hydrated images in Fig. 5. (ZIP)
